# Cognitive psychology: computing the value of the choices we do not make

**DOI:** 10.1038/s44271-024-00064-x

**Published:** 2024-03-02

**Authors:** Inti A. Brazil

**Affiliations:** Donders Institute for Brain, Cognition, and Behaviour, Radboud University Nijmegen, Nijmegen, Netherlands

## Abstract

Reflecting on choices we did make and those we could have made is very common. In a recent study in *Science Advances*, researchers used a reinforcement learning paradigm together with computational modeling to study the processes underlying the value update of unchosen actions.

**Figure:**
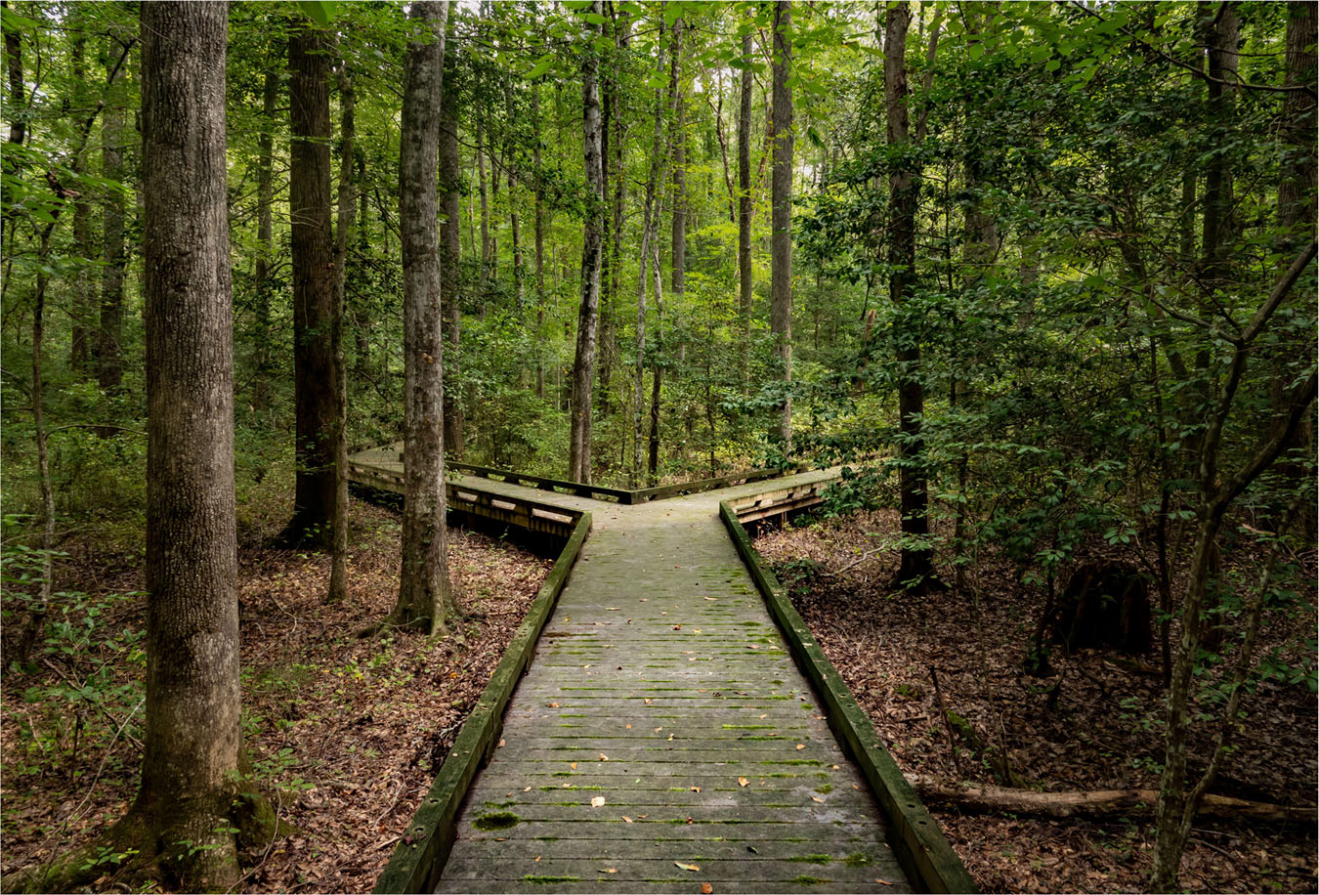
BackyardBest for Alamy

Research on decision-making suggests that we use feedback to assign values to different choice options. We tend to act in ways that are routinely associated with favorable outcomes and avoid behaviors leading to poor outcomes. But how do we learn the value of unchosen options for which we do not obtain feedback, given that they represent hypothetical scenarios that never occurred?

A recent study by Ben-Artzi and colleagues^1^ moves us closer towards obtaining an answer. Participants performed a multi-armed bandit task, in which they were presented with 2 (out of 4 cards) on each trial and had to learn which card to choose in order to obtain the highest monetary gain. By combining a clever task-design with the use of computational models, they were able to estimate the value assigned to cards that were not chosen and elucidate a possible underlying mechanism.

One key finding was that the values of the unchosen options were updated relative to the value of the chosen option, by integrating the history of outcomes for choosing a particular card with that of rejecting the other cards. The findings suggest that choosing an option that leads to a favorable outcome may reinforce the avoidance of alternative choices, and the value of each unchosen option is updated even when this is not required to perform the task.

Such discoveries could inspire studies in (patient) populations associated with disturbances in reinforcement-based decision-making. For example, selective reinforcement learning impairments have been observed in offenders with strong psychopathic tendencies (e.g., callousness, recklessness)^2^, who often fail to avoid choices that previously led to rewards but no longer do. Ben-Artzi et al.’s^1^ results could suggest that, rather than failing to update information pertaining to the choice made, such individuals perhaps fail to detect when alternative choices have become more favorable and, therefore, keep making poor choices.

This study sheds new light on the mechanisms of counterfactual thinking and could also new avenues for research in other subfields of psychology.
